# Effects of Peripheral Neural Blocks in Laparoscopic Sleeve Gastrectomy: a Pilot Study on Cognitive Functions in Severe Obese Patients

**DOI:** 10.1007/s11695-022-06319-y

**Published:** 2022-11-05

**Authors:** Xinyang Zhao, Qi Xue, Ling Dong, Zhaoxia Chu, Yong Wang, Chanjuan Chen, Xianwen Hu, Ye Zhang, Chunxia Huang

**Affiliations:** 1 Department of Anesthesiology and Perioperative Medicine, the Second Affiliated Hospital of Anhui Medical University, Hefei City, 230000 Anhui Province China; 2 Key Laboratory of Anesthesiology and Perioperative Medicine of Anhui Higher Education Institutes, Anhui Medical University, Hefei City, 230000 Anhui Province China; 3 Department of Anesthesiology, The First Affiliated Hospital of Anhui University of Chinese Medicine, Hefei City, 230000 Anhui Province China; 4 Department of General Surgery, the Second Affiliated Hospital of Anhui Medical University, Hefei City, 230000 Anhui Province China; 5 Department of Oncology, the Second Affiliated Hospital of Anhui Medical University, Hefei City, 230000 Anhui Province China

**Keywords:** Transverse abdominis plane block, Quadratus lumborum block, Cognition, Inflammation, Laparoscopic sleeve gastrectomy

## Abstract

**Background:**

In addition to the analgesic effect, peripheral neural blocks also prevent cognitive impairment and peripheral inflammation induced by surgery. However, it is unknown if there is collateral impact on cognitive improvement after bariatric surgery.

**Methods:**

In this pilot study, 75 patients with severe obesity for selective laparoscopic sleeve gastrectomy (LSG) were recruited and randomized into three groups (1:1:1) as general anesthesia (GA) group, transverse abdominis plane block (TAPB) group, and quadratus lumborum block (QLB) group. Bilateral TAPB or QLB was performed (0.33% ropivacaine with dexmedetomidine 1 μg/kg) before the standardized general anesthesia. Cognitive test battery was completed before LSG and in 1-month and 3-month follow-up. The levels of peripheral inflammatory cytokines were determined at equivalent time points.

**Results:**

Patients with LSG exhibited massive cognitive improvement in postoperative 3 month without or with TAPB or QLB (*P*_time_ < 0.001). Compared to GA, QLB significantly strengthened performance in MoCA (*β* = 0.56, 95%CI: 0.08, 1.05). IL-6, IL-8, and high-sensitivity CRP significantly verified among three groups. Changes in IL-6 within postoperative 3 months were negatively correlated with MMSE and MoCA, and positively correlated with AVLT-DR for QLB group. Similar correlation was found in the GA group for changes in IL-6 and AVLT-IR.

**Conclusion:**

Laparoscopic sleeve gastrectomy ideally improved memory and attention as early as postoperative 1 month. QLB promoted cognitive improvement in MoCA, which was negatively correlated with changes in IL-6. More precise trials are needed to determine the overall effect of peripheral neural block on cognition following bariatric surgery.

**Graphical Abstract:**

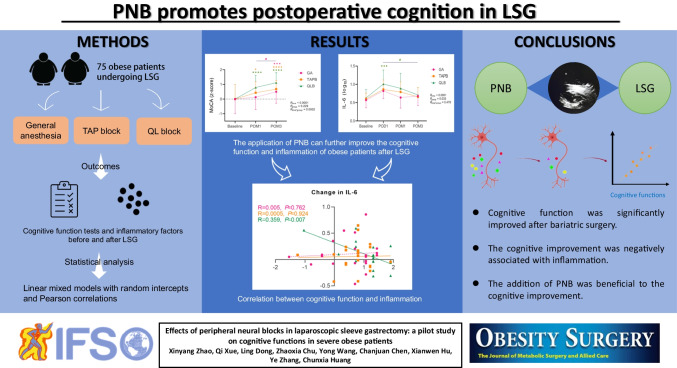

**Supplementary Information:**

The online version contains supplementary material available at 10.1007/s11695-022-06319-y.

## Introduction

According to the definition by WHO, overweight is a body mass index (BMI) ≥ 25 kg/m^2^, and obesity is a BMI ≥ 30 kg/m^2^. Obesity is a major public health concern throughout the world among children and adolescents, as well as adults. The prevalence of obesity has tripled over the past four decades, and there will be 1 billion obese adults by 2025. The burden in enormous medication, society, and economics induced by obesity is highly associated with series of subsequent diseases including diabetes mellitus, cardiovascular disease, sleep-breathing disorders, musculoskeletal disorders, and certain forms of cancer [1, 2]. Meanwhile, obesity is negatively correlated with various cognitive functions such as individual attention, memory, executive function, decision-making ability, and language learning, and this negative correlation exists in all age stages. The metabolic dysfunction, dyslipidemia, and inflammation caused by obesity contribute to the development of numerous disorders in the nervous system [3]. In particular, the secondary neuroinflammation is focused not only on the hypothalamus, but also on hippocampus, cortex, brainstem, or amygdala [4]. Therefore, pharmacological and invasive surgery has received tremendous attention to treat obesity.

Bariatric surgery is a promising intervention for people with severe obesity. It profoundly improves the comorbidities and reduces the overall mortality during the long-term follow-up [5, 6]. The improved neurological function may attribute to the improvement in adipokine [7], insulin [8], and inflammation signaling [9]. While memory performance is improved 12 months after bariatric surgery, the mechanisms underlying these improvements have not been fully understood. It is not associated with optimal postsurgical variations, including reductions in BMI or comorbid medical conditions [10].

To efficiently relieve postoperative pain, peripheral neural blocks have been applied in the multimodal analgesia management for bariatric surgery. Evidence suggests that transverse abdominis plane block (TAPB) and quadratus lumborum block (QLB) can significantly reduce postoperative pain following abdominal surgery [11, 12]. In addition, TAPB and QLB also prevent peripheral inflammation and cognitive impairment induced by surgery [13, 14]. However, few studies have evaluated the impact of TAPB and QLB on cognition after bariatric surgery. In our previous study, TAPB and QLB provided comparable pain relief in laparoscopic sleeve gastrectomy (LSG). To further explore if there was additional effect on cognition and peripher inflammation during the follow-up, the pilot study was conducted in the obesity population underwent LSG.

## Materials and Methods

### Participants

This is a pilot study based on the effects of peripheral neural block (PNB) in cognitive improvement among severe obese patients after laparoscopic sleeve gastrectomy (LSG). The study design was described previously, one of the primary aims of this RCT was to compare the analgesic effect of TAPB and QLB during LSG [12]. All participants were randomized into following three groups: (1) general anesthesia (group GA); (2) general anesthesia plus transversus abdominis plane block (TAPB) (group TAPB); and (3) general anesthesia plus quadratus lumborum block (QLB) (group QLB). This RCT was approved by the hospital’s ethics committee. Informed consent was obtained from all individual participants included in the study. All demographic and clinical characteristics were collected with case report form by a doctor who was blinded to the randomization and interventions. From the total RCT population, there were 65 patients who completed both cognitive evaluation and peripheral blood collection, and then included in the present analysis.

### Standardized Anesthesia Protocol

Before anesthesia, all patients were delivered to the pre-anesthesia room for the arterial intubation and ultrasound-guided PNB. In brief, TAPB and QLB were performed by an experienced anesthesiologist who was blinded to the trial. By using a convex array probe (Sonosite Micromaxx, Bothell, WA, USA) with a frequency of 2–5 MHz, bilateral TAPB or QLB was completed with the diffusion of 30 ml of 0.33% ropivacaine (including dexmedetomidine 1 ug/kg) or normal saline into the fascia plane.

A standardized anesthesia protocol was conducted to all patients. Briefly, perioperative administration of dexmedetomidine was achieved by a bolus infusion at 2 μg/kg/h for 15 min and a continuous infusion at 0.4 μg/kg/h during the surgery, as well as the subsequent patient-controlled intravenous analgesia (PCIA) device after surgery. General anesthesia induction was accomplished with midazolam (0.025 mg/kg), propofol (1.5 mg/kg), cisatracurium (0.2 mg/kg), and remifentanil (2 μg/kg). General anesthesia was maintained with sevoflurane inhalation as well as continuous infusion of remifentanil (5–15 μg/kg/h) and cisatracurium (0.1–0.2 mg/kg/h). The doses of all intravenous anesthetics were calculated based on the ideal body weight for each patient. During the operation, vasoactive agents were applied to maintain the mean arterial pressure (MAP) and heart rate (HR) within a range of 20% more or less than the baseline. Parecoxib 40 mg and nalbuphine 10 mg were also used as analgesia complement. The same surgeon team performed all the surgical procedures by using five ports technique.

### Cognitive Evaluation

To continuously evaluate patients’ cognition, a battery of clinical neuropsychological assessments was used before LSG, 1 month and 3 months after LSG. The cognitive battery consisted of Montreal Cognitive Assessment (MoCA) [15], Mini-mental State Examination (MMSE) [16], Auditory Verbal Learning Test (AVLT) [17], Stroop Color Word Test (SCWT) [18], and Digital Span Test (DST) [19]. MMSE and MoCA were used to assess the global cognitive function. AVLT mainly reflects the memory function. SCWT is one of the golden standards of attentional measures. DST indicates working memory function. There were two indicators in SCWT including completion time and accuracy in three independent sessions. The average values of completion time were included in the final analysis. Higher scores indicated better performance on all assessments, except for SCWT, in which higher scores indicated worse performance. All tests were conducted by a doctor who received standard training and was blinded to the randomization and interventions for all participants.

### Inflammatory Cytokines Assessment

Plasma was collected before surgery, 24 h after surgery, 1 month after surgery, and 3 months after surgery. The concentrations of TNF-*α*, IL-6, IL-8, IL-1*β*, IL-10, MCP-1, CRP, and BDNF in plasma were determined using a customized Milliplex Human Cytokine Immunoassay Kits (Millipore, USA) with Analyzer 3.1 Luminex 200 machine, and analyzed on corresponding software according to the manufacturer’s instructions. Values were referenced to a standard curve generated from eight calibrators with known concentrations. Plasma was diluted according to the standard curve, and values were converted to log10 in the final analysis.

### Statistical Analysis

Baseline characteristics of three groups were described as mean ± standard deviation (SD), median with interquartile ranges or numbers (percentages), which were analyzed with one-way analysis of variance, *chi*-square tests, or Kruskal–Wallis H tests as appropriated. Raw scores for all cognitive assessments were standardized with baseline scores to generate relative *z*-scores. A higher *z*-score means better performance, except for SCWT, which denotes a worse performance. The values of inflammatory cytokines were log_10_-transformed before analysis, due to the abnormal distribution. Linear mixed models with random intercepts were used to evaluate the changes in cognitive function and inflammatory cytokines in postoperative 3 months. All models were adjusted for age, sex, BMI, hypertension, hyperglycemia, and hyperlipidemia at baseline. Cognitive performance was further adjusted for the education year. Multiple comparisons with Šidák adjustment were conducted to further investigate the changes in cognitive functions and inflammatory cytokines at different time points and in different groups. Pearson correlations were conducted to test the association between changes in inflammatory cytokines and standard cognitive scores in postoperative 3 months. All analyses were performed with SAS version 9.4 (SAS Institute Inc.). Two-sided *P* values less than 0.05 were considered as statistically significant.

## Results

**Fig. 1:**
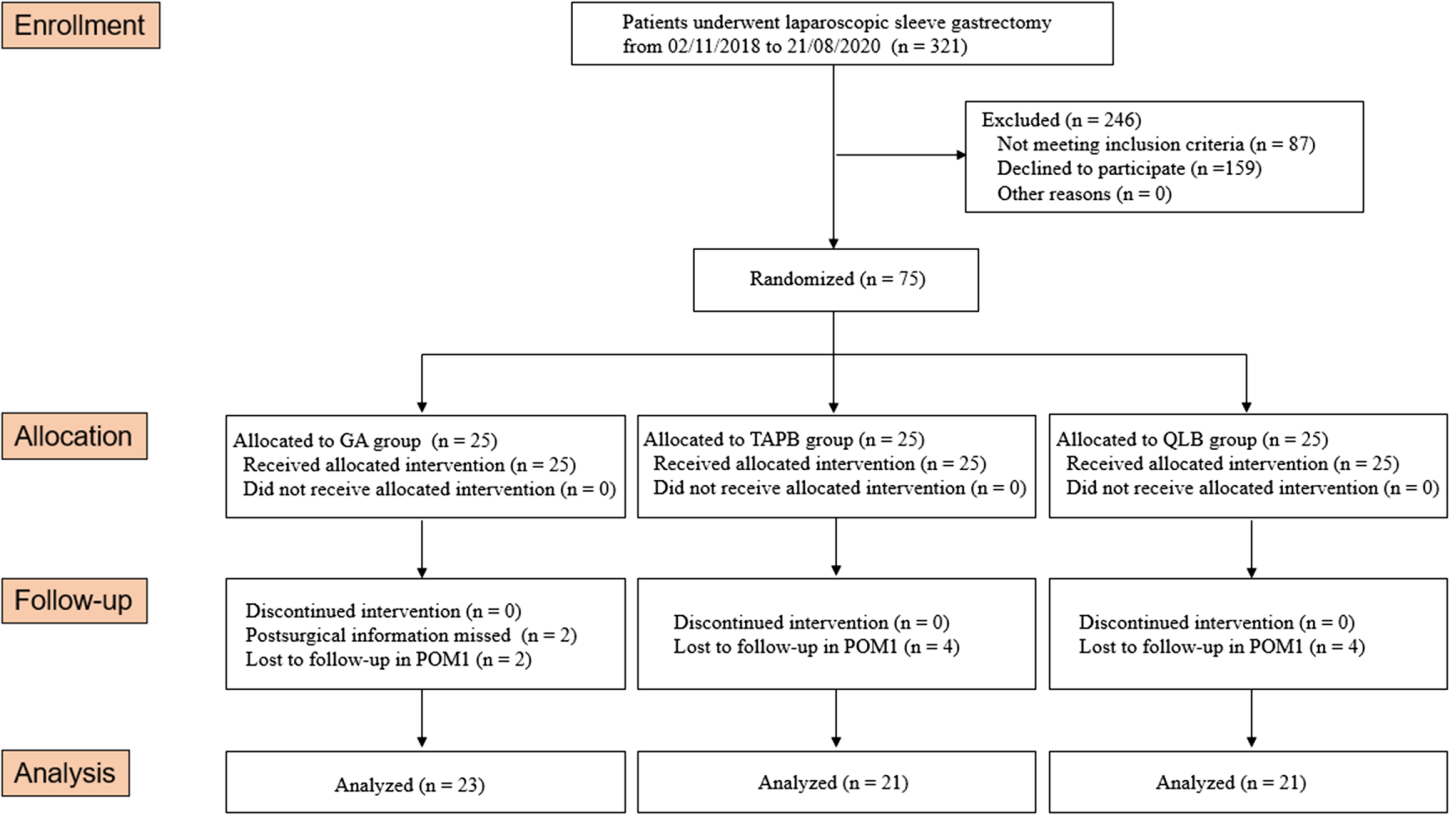
Flowchart of the pilot study. POM, postoperative month

Among 75 patients included in this pilot study, there were 10 patients lost to follow-up in the first month after surgery. In the final analysis, 23 patients received GA, 21 had TAPB, and another 21 received QLB. Before and after LSG, 65 obese patients completed cognitive battery and plasma levels of inflammatory cytokines at different time points (Fig. 1). No significant differences were observed among three groups in basic demographic and clinical characteristics (Table 1).

**Table 1:** Comparison of demographic and clinical characteristics between groups

		GA (*n* = 23)	TAPB (*n* = 21)	QLB (*n* = 21)	*P* value
Age		31.0 (25.0, 34.0)	29.0 (25.5, 35.0)	33.0 (23.5, 39.0)	0.960
Sex/male		9 (39.1%)	7 (33.3%)	7 (33.3%)	0.897
BMI		43.2 (39.2, 45.7)	42.1 (37.5, 45.0)	46.7 (38.6, 51.0)	0.510
Education year		15.0 (12.0, 15.0)	15.0 (12.8, 16.0)	15.0 (12.0, 15.5)	0.240
ASA					0.653
	II	20 (87.0%)	16 (76.2%)	17 (81.0%)	
	III	3 (13.0%)	5 (23.8%)	4 (19.0%)	
Hypertension		15 (65.2)	15 (71.4)	14 (66.7)	0.901
Hyperglycemia		15 (65.2)	13 (61.9)	14 (66.7)	0.947
Hyperlipidemia		16 (69.6%)	20 (95.2%)	18 (85.7%)	0.062
OSAHS		22 (95.7%)	18 (85.1%)	21 (100%)	0.100
POCS		3 (13.0%)	2 (9.5%)	4 (19.0%)	0.667
Arthritis		1 (4.3%)	3 (14.3%)	4 (19.0%)	0.274

Data were expressed as median (*P*_25_, *P*_75_) or numbers and percentages. *P* values were calculated by Kruskal–Wallis *H* tests and Chi-square tests as appropriate. *OSAHS*, obstructive sleep apnea hypopnea syndrome; *POCS*, polycystic ovary syndrome

**Fig. 2:**
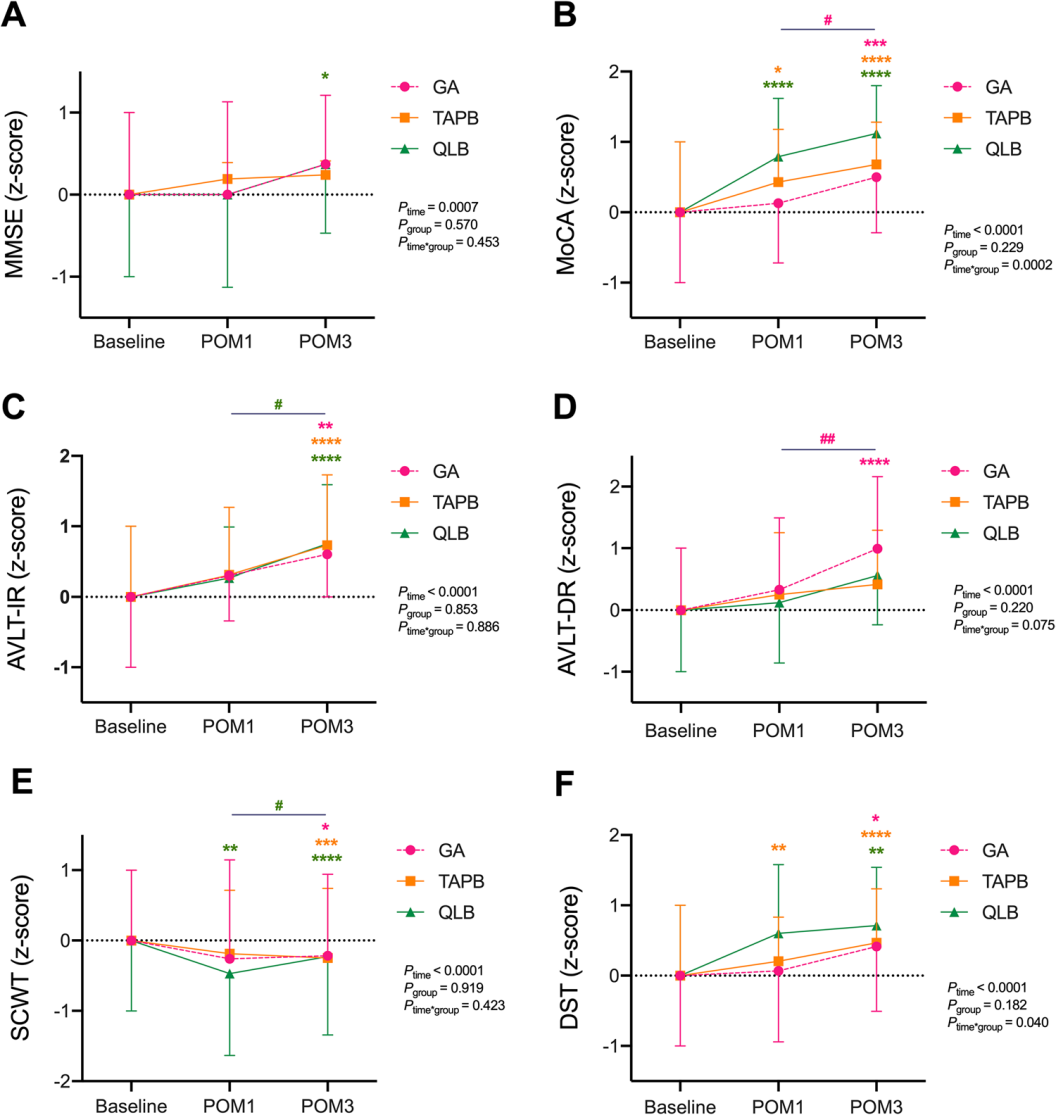
Changes of cognitive functions among patients underwent laparoscopic sleeve gastrectomy. ^*^vs. Baseline in the same group. ^*^P < 0.05, ^**^P < 0.01, ^***^P < 0.001, ^****^P < 0.0001; ^#^*P* < 0.05, ^##^*P* < 0.01. POM, postoperative month; MMSE, Mini-mental State Examination; MoCA, Montreal Cognitive Assessment; AVLT-IR, Auditory Verbal Learning Test-Immediate Recall; AVLT-DR, Auditory Verbal Learning Test-Delayed Recall; SCWT, Stroop Color Word Test; DST, Digital Span Test

The correlation matrix for raw data between different cognitive tests at each time point was reported in Fig. S1. The main effects of time revealed a significant improvement of all cognitive functions after LSG (*P* < 0.001) (Fig. 2). When comparing with baseline by using multiple comparisons, MoCA scores were consistently increased in both TAPB and QLB groups in postoperative month (POM) 1. At the same time, improved cognition indicated by lower SCWT scores and higher DST scores was presented in QLB group and TAPB group, respectively. When comparing with POM1, patients in GA group performed better in MoCA and AVLT-DR in POM3 (*P* < 0.05). In QLB group, AVLT-IR score was significantly increased in POM3 comparing with POM1, which suggested that immediate memory was improved. There were significant group × time interaction effects in MoCA and DST (*P*_interaction for MoCA_ = 0.0002, *P*_interaction for DST_ = 0.040).

The linear mixed models were used to demonstrate between-group differences in cognitive functions by comparing with GA group (Table 2). Significant increases of MoCA (*β* = 0.56, 95%CI: 0.08, 1.05, *P* = 0.023) indicated the enhanced cognitive improvement induced by QLB. However, significant decreases in AVLT-DR scores were found in both TAPB group (adjusted *β* =  − 0.77; 95%CI: − 1.34, − 0.19; *P* = 0.010) and QLB group (adjusted *β* =  − 0.59; 95%CI: − 1.15, − 0.03; *P* = 0.040), which implied the weakened cognition.

**Table 2:** Linear mixed effects in cognitive functions

	GA	TAPB	QLB
MMSE			
* β* (95%CI)	ref	− 0.23 (− 0.75, 0.30)	0.23 (− 0.27, 0.74)
* P*		0.385	0.355
MoCA			
* β* (95%CI)	ref	0.13 (− 0.38, 0.64)	**0.56 (0.08, 1.05)**
* P*		0.611	**0.023**
AVLT-IR			
* β* (95%CI)	ref	− 0.04 (− 0.58, 0.50)	0.07 (− 0.45, 0.58)
* P*		0.891	0.790
AVLT-DR			
* β* (95%CI)	ref	− **0.77 (**− **1.34,** − **0.19)**	− **0.59 (**− **1.15,** − **0.03)**
* P*		**0.010**	**0.040**
SCWT			
* β* (95%CI)	ref	0.02 (− 0.56, 0.60)	− 0.15 (− 0.70, 0.39)
* P*		0.944	0.576
DST			
* β* (95%CI)	ref	0.44 (− 0.07, 0.94)	0.10 (− 0.38, 0.58)
* P*		0.089	0.669

Models were analyzed using *z*-scores and adjusted by age, sex, BMI, hypertension, hyperglycemia, hyperlipidemia, and education year at baseline

*MMSE*, Mini-mental State Examination; *MoCA*, Montreal Cognitive Assessment; *AVLT-IR*, Auditory Verbal Learning Test-Immediate Recall; *AVLT-DR*, Auditory Verbal Learning Test-Delayed Recall; *SCWT*, Stroop Color Word Ttest; *DST*, Digital Span Test; *CI*, confidence interval

LSG could also influence the plasma levels of pro-inflammatory cytokines and BDNF (*P*_time_ < 0.001, Fig. S2 and Table 3), except the anti-inflammatory IL-10. Šidák-corrected comparisons indicated the increases of TNF-*α* and CCL2 at (postoperative day) POD1 in GA group, which was significantly reduced to preoperative levels in POM3. In TAPB group, hs-CRP level at POD1 was significantly decreased, and BDNF was significantly increased in POM1 and POM3. Otherwise, patients in QLB group had lower levels of TNF-*α*, IL-6, CCL2, and IL-8 and higher BDNF in POM3. There was a significant group × time interaction for TNF-*α*, hs-CRP, and BDNF. In particular, hs-CRP in TAPB group was significantly lower than that in GA group at POD1 (*P* < 0.001). Moreover, compared to the GA group, patients with TAPB treatment had a significant higher IL-8 (*β* = 0.18, 95%CI: 0.04, 0.32, *P* = 0.011), and those in QLB group had higher BDNF (*β* = 0.15, 95%CI: 0.01, 0.29, *P* = 0.038) (Table S1).

**Table 3:** Comparison of inflammatory cytokines in different groups at different timepoints

		GA group			TAPB group	QLB group		*P*_time_	*P*_group_	*P*_interaction_	
TNF-*α*								** < 0.001**	0.893	**0.006**	
	Baseline	0.71 ± 0.20			0.75 ± 0.15	0.77 ± 0.20					
	POD1	1.00 ± 0.21			0.84 ± 0.23	0.88 ± 0.21					
	POM1	0.71 ± 0.19			0.73 ± 0.20	0.76 ± 0.19					
	POM3	0.69 ± 0.16			0.76 ± 0.14	0.64 ± 0.16					
IL-1*β*								**0.001**	0.542	0.315	
	Baseline	0.70 ± 0.14			0.73 ± 0.13	0.78 ± 0.14					
	POD1	0.88 ± 0.24			0.80 ± 0.21	0.83 ± 0.24					
	POM1	0.74 ± 0.19			0.82 ± 0.21	0.83 ± 0.19					
	POM3	0.71 ± 0.15			0.74 ± 0.12	0.68 ± 0.15					
IL-6								** < 0.001**	**0.033**	0.478	
	Baseline	0.57 ± 0.21			0.60 ± 0.16	0.63 ± 0.21					
	POD1	0.82 ± 0.21			0.86 ± 0.26	1.01 ± 0.21					
	POM1	0.64 ± 0.29			0.79 ± 0.28	0.89 ± 0.29					
	POM3	0.68 ± 0.26			0.66 ± 0.25	0.71 ± 0.26					
IL-8								** < 0.001**	**0.048**	0.385	
	Baseline	0.54 ± 0.14			0.66 ± 0.14	0.65 ± 0.14					
	POD1	0.71 ± 0.36			0.73 ± 0.22	0.82 ± 0.36					
	POM1	0.56 ± 0.23			0.69 ± 0.21	0.68 ± 0.23					
	POM3	0.45 ± 0.18			0.64 ± 0.23	0.57 ± 0.18					
CCL2								** < 0.001**	0.132	0.092	
	Baseline	2.19 ± 0.22			2.26 ± 0.17	2.33 ± 0.22					
	POD1	2.38 ± 0.16			2.36 ± 0.17	2.40 ± 0.16					
	POM1	2.29 ± 0.20			2.30 ± 0.14	2.34 ± 0.20					
	POM3	2.20 ± 0.22			2.27 ± 0.16	2.20 ± 0.22					
IL-10								0.307	0.169	0.270	
	Baseline	0.41 ± 0.21			0.36 ± 0.21	0.37 ± 0.21					
	POD1	0.37 ± 0.20			0.28 ± 0.15	0.41 ± 0.20					
	POM1	0.30 ± 0.17			0.29 ± 0.21	0.42 ± 0.17					
	POM3	0.37 ± 0.17			0.38 ± 0.18	0.43 ± 0.17					
hs-CRP								**0.009**	**0.040**	**0.005**	
	Baseline	4.63 ± 0.11			4.57 ± 0.11	4.57 ± 0.11					
	POD1	4.67 ± 0.11			4.45 ± 0.17	4.54 ± 0.11					
	POM1	4.61 ± 0.17			4.51 ± 0.13	4.53 ± 0.17					
	POM3	4.53 ± 0.20			4.51 ± 0.14	4.52 ± 0.20					
BDNF								** < 0.001**	0.208	**0.022**	
	Baseline	3.48 ± 0.20			3.41 ± 0.24	3.50 ± 0.20					
	POD1	3.37 ± 0.22			3.32 ± 0.29	3.46 ± 0.22					
	POM1	3.45 ± 0.21			3.56 ± 0.29	3.58 ± 0.21					
	POM3	3.50 ± 0.23			3.60 ± 0.25	3.64 ± 0.23					

*POD*, postoperative day; *POM*, postoperative month

Data in boldface were statistically significant (*P* < 0.05)

To test the association between varied inflammatory cytokines and standardized cognitive scores, Pearson correlation analysis was applied in postoperative 3 months. In the GA group, there was a significant positive correlation between MMSE and changes in IL-8 (*r* = 0.534, *P* = 0.015) and CCL2 (*r* = 0.612, *P* = 0.004), as well as a negative correlation between DST and change in IL-1*β* (*r* =  − 0.539, *P* = 0.014). In the TAPB group, positive correlations were found between MoCA and changes in hs-CRP (*r* = 0.467, *P* = 0.038), DST and changes in CCL2 (*r* = 0.490, *P* = 0.028), respectively. Negative correlations between MMSE and changes in BDNF (*r* =  − 0.461, *P* = 0.041), AVLT-IR and changes in BDNF (*r* =  − 0.512, *P* = 0.021) were also reported after LSG with TAPB. For QLB group, changes in IL-6 had a significant negative correlation with MMSE (*r* =  − 0.496, *P* = 0.031) and MoCA (*r* =  − 0.599, *P* = 0.007), respectively, and a positive correlation with AVLT-DR (*r* = 0.642, *P* = 0.007) (Fig. S3).

## Discussion

In this study, we successfully described the profiles of cognition and inflammation before and after bariatric surgery. Among adults with obesity, cognition was significantly improved in 3 months after LSG. QLB, as an adjunct to the general anesthesia, significantly promoted cognitive improvement in MoCA. Significant reductions of pro-inflammatory cytokines and an increase of BDNF were also observed during the postoperative follow-up. There were possible correlations between cognition and changes in peripheral inflammatory cytokines, especially pro-inflammatory cytokines in postoperative 3 months.

Obesity itself is sufficient to impair cognition [20]. According to the current evidence of analyzing 72 studies with 4904 overweight/obese participants, there were a broad of deficits in executive function in obese individuals, and inhibition and working memory deficits in overweight individuals [21]. Fortunately, numbers of longitudinal studies and RCTs support that bariatric surgeries rapidly improve cognitive deficits in attention, memory, executive function, and language when achieving profound weight loss [22]. There is convincing evidence that 1 year after surgery, cognitive improvements were still presented in both attention and executive function, which were evidenced by Digit Span Total, Switching of Attention and Verbal Interference [23]. These improvements would persist even several years [24], and may be associated with structural reorganization of brain induced by bariatric surgery [25], such as greater white matter and gray matter integrity during the long-term follow-up [26]. In our study of all 65 patients, there was consistently better performance in MoCA and SCWT in postoperative 1 month than baseline. Moreover, significantly better performances were broadly presented in MoCA, SCWT, AVLT-IR, and DST in postoperative 3 months rather than 1 month. The significant cognitive improvements induced by bariatric surgery presented in memory and attention.

On the other hand, according to the analysis of 122 studies from 1946 to 2020, PNB is associated with decreased occurrence of numerous complications after total hip and knee arthroplasties, such as cognitive dysfunction, respiratory failure, and cardiac complications [27]. In particular, QLB improves the declines in MMSE and MoCA scores in elderly patients in laparoscopic radical gastrectomy [14]. Overall, we found that the increased cognitive scores displayed in the majority of cognition battery after 3 months in those patients with QLB. As the addition to standard general anesthesia protocol, QLB profoundly increased MoCA score (*β* = 0.56, *P* = 0.023) comparing with general anesthesia alone. After LSG with either TAPB or QLB, the cognitive improvement with higher MoCA scores was presented as early as postoperative 1 month. However, delayed recall memory was improved after general anesthesia rather than TAPB or QLB treatment in AVLT test.

Chronic adipose tissue inflammation and associated increases of circulating pro-inflammatory adipokines are typically displayed in patients with obesity. Inflammation has been regarded as a critical mechanism of cognitive dysfunction in human [9] and animal models of obesity [28], evidenced by the accumulation of proinflammatory cytokines (TNF-*α*, IL-1*β*, and IL-6) [29–31]. The higher body mass index (BMI), the worse cognitive function. The major contributor is the increased peripheral IL-6 [32]. Elevated IL-6 but not CRP in midlife prospectively predicts cognitive decline, evidenced by MMSE [33]. The modulation of the cognitive performance induced by bariatric surgery is independent on the type of surgical procedure [25]. Variations of adipokines, gut hormones, and gut microbiota induced by bariatric surgery partially contributed to the rapid and persistent neurological modification, which was associated with weight loss [26]. The long-term metabolic improvements following surgery include increased circulating adiponectin, decreased secretion of pro-inflammatory interleukins (1, 6, and 8), and leptin secretion [34]. We found that levels of pro-inflammatory cytokines (TNF-*α*, IL-1*β*, IL-6, IL-8, CCL2, and hs-CRP) significantly dropped in postoperative 3 months. The impact of TAPB on inflammation was presented by the significant decrease of hs-CRP at postoperative 24 h. As the primary signature of peripheral immune activation, elevated IL-6 is indeed directly released by adipocytes. Although CRP is a sensitive biomarker of chronic low-grade inflammation and is associated with multiple complex diseases, CRP is of hepatic origin and released secondary to IL-6 [35, 36]. Both higher white matter hyperintensities (WMH) and higher BMI contributed to increased deep-to-periventricular WMH ratio through elevated IL-6 rather than CRP, although CRP was in the similar tendency [10]. Furthermore, decreases in hs-CRP are also not associated with postoperative cognitive benefits in 1 year after bariatric surgery [23]. We also found a significant negative correlation between MoCA scores and IL-6 levels in surgical patients with QLB in 3-month follow-up. However, the unchanged IL-6 level may due to contradictory impact of bariatric surgery on IL-6 in obese patients. Postsurgical IL-6 level is even higher than the baseline with QLB intervention [37].

Brain-derived neurotrophic factor (BDNF), as a member of the neurotrophin family, originally supports the development, maintenance, and plasticity of the central and peripheral nervous systems. BDNF signaling also contributes to the development of the metabolic syndrome and obesity under a circumstance with chronic positive energy balance. At the same time, the reduction of BDNF enforces the susceptibility to Alzheimer’s disease and other age-related neurodegenerative disorders [38]. Genetic variation of BDNF and the reduced protein expression of BDNF are associated to obesity in both human and animal models. However, there are similar levels of BDNF between patients and control in a meta-analysis [39] and a RCT [40]. After Roux-en-Y gastric bypass, BDNF levels decreased from 15.3 ± 1.02 ng/ml to 11.93 ± 0.96 ng/ml in postsurgical 1 year [40]. In bariatric surgery, the altered energy balance may contribute to the reduced circulating BDNF and rapid weight loss. Periphery BDNF is decreased from preoperative 7708 pg/ml to postoperative 4146.3 pg/ml in women [41]. Otherwise, the levels of BDNF in our study were much lower than reported, and elicited significant correlation with MMSE in postoperative 3 months.

Noteworthy, the addition of dexmedetomidine may partially participate in the changes of cognition and inflammation induced by TAPB or QLB in this study. Dexmedetomidine is a selective *α*2-adrenergic receptor agonist with multiple biomedical behaviors. In elderly patients admitted to the intensive care unit after noncardiac surgery, low-dose dexmedetomidine infusion significantly decreased postoperative delirium within 1 week. Long-term outcome is demonstrated in 3-year survivors from the same population, in which patients with dexmedetomidine infusion achieved better cognitive performance and quality of life than that with placebo [42]. The addition of dexmedetomidine to PNB (e.g., thoracic nerve block) enhances recovery after thoracic surgery, which may be mediated by attenuating lung injury and cellular immune dysfunction [43]. Dexmedetomidine plays a supplementary role in prolonging analgesia duration and enhancing analgesic effect of PNB. In addition, it reduces the surgical stress and ultimately affects postoperative cognition [44].

There are also some limitations in our study. Some bias might affect inflammatory cytokines assessments, especially BDNF measurements, such as physical exercise [45] and diet control [46] after surgery. The sample size was rather small with mid-term follow-up, and further studies will include a larger population with longer follow-up to indicate the effect of PNB on cognitive function.

## Conclusion

In sum, our clinical trial suggested that the bariatric surgery effectively improved the cognitive function and modified the inflammation in obesity patients. The application of regional neural block in the standardized general anesthesia could further improve patient’s memory and attention. Numerous pro-inflammatory cytokines and BDNF may contribute to the massive cognitive improvements. Therefore, obesity treatment should be individually tailored with multiple analgesic approaches. Our findings provided the first clue to what impact of PNB on cognition.

## Supplementary Information

Below is the link to the electronic supplementary material.

Supplementary file1 (DOCX 37810 KB)
