# Supplementary material for: Effects of Peripheral Neural Blocks in Laparoscopic Sleeve Gastrectomy: a Pilot Study on Cognitive Functions in Severe Obese Patients
Source: Obes Surg. 2022 Nov 5;33(1):129–38. doi: 10.1007/s11695-022-06319-y (PMC9834365; doi:10.1007/s11695-022-06319-y)

**Supplementary information**

**Fig. S1 Correlation matrix between cognitive assessments at each time point.**

**Fig. S2 Variations in inflammatory cytokines in patients underwent laparoscopic sleeve gastrectomy.**

**Table S1 Linear mixed effect in inflammatory cytokines**

**Fig. S3 Correlation plots of association between changes in inflammatory cytokines and standardized cognitive scores in postoperative 3 months**


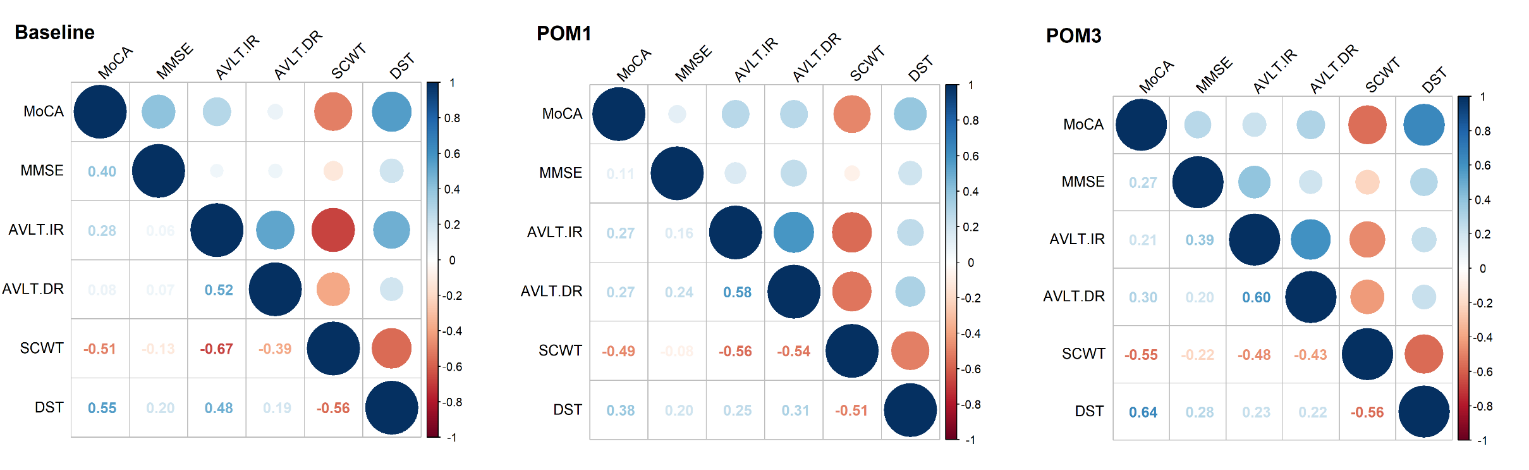


**Fig. S1 Correlation matrix between cognitive assessments at each time point.**

POM, postoperative month; MMSE, Mini-mental State Examination; MoCA, Montreal Cognitive Assessment; AVLT-IR, Auditory Verbal Learning Test-immediate recall; AVLT-DR, Auditory Verbal Learning Test-delayed recall; SCWT, Stroop Color Word Test; DST, Digital Span Test.

**
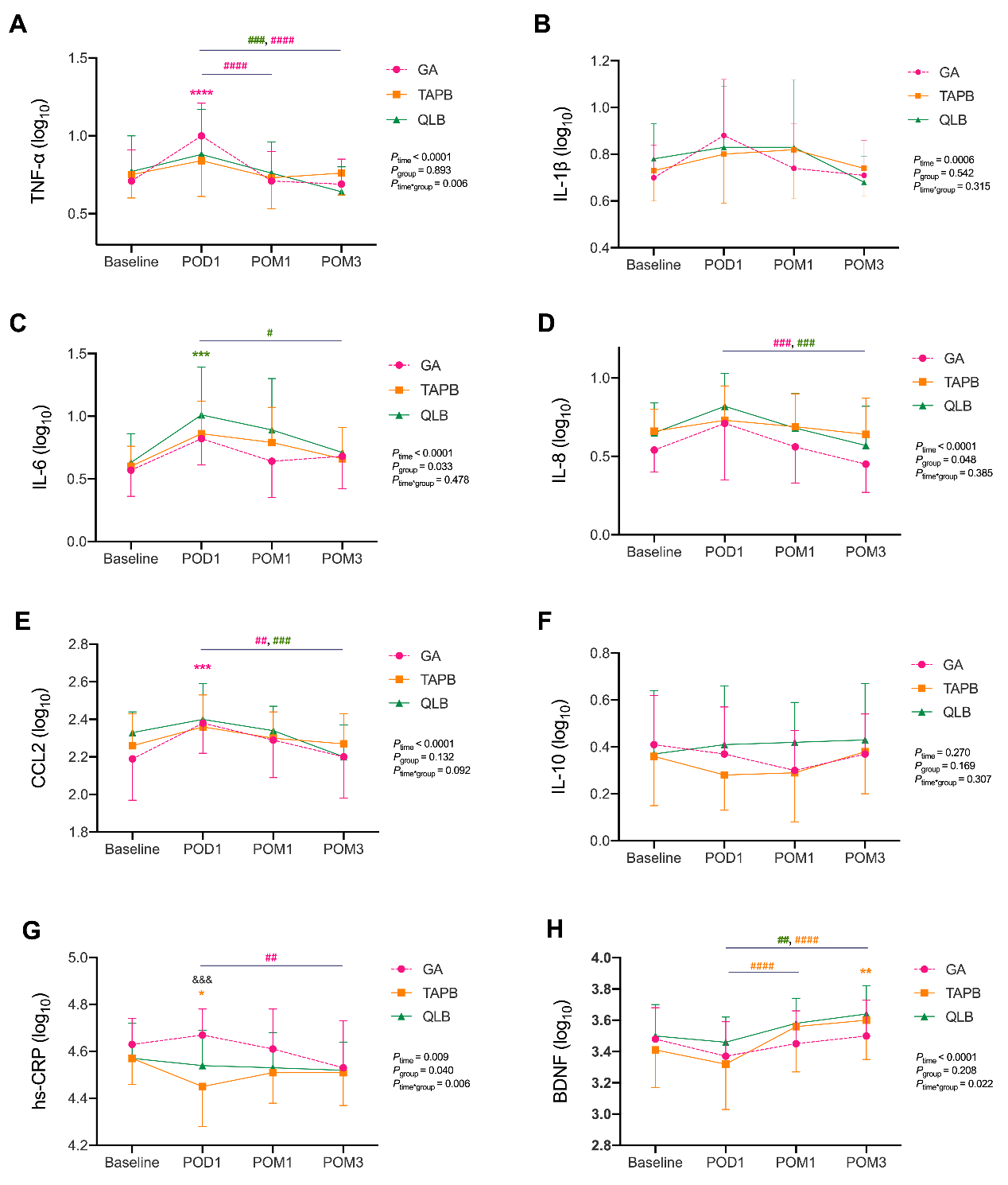
**

**Fig. S2 Variations in inflammatory cytokines in patients underwent laparoscopic sleeve gastrectomy.**

^*^vs. Baseline in the same group; ^&^TAPB vs. GA at the time point. ^*^*P* < 0.05, ^**^*P* < 0.01, ^***^*P* < 0.001, ^****^*P* < 0.0001; ^##^*P* < 0.01, ^###^*P* < 0.001, ^####^*P* < 0.0001; ^&&&^*P* < 0.001.

POD, postoperative day; POM, postoperative month; MMSE, Mini-mental State Examination; MoCA, Montreal Cognitive Assessment; AVLT-IR, Auditory Verbal Learning Test-immediate recall; AVLT-DR, Auditory Verbal Learning Test-delayed recall; SCWT, Stroop Color Word Test; DST, Digital Span Test.

| **Table S1 Linear mixed effect in inflammatory cytokines** | | | | |
| --- | --- | --- | --- | --- |
|  | | GA | TAPB | QLB |
| TNF-α | |  |  |  |
|  | β (95%CI) | ref | 0.06 (-0.07, 0.19) | -0.06 (-0.19, 0.08) |
|  | *P* |  | 0.381 | 0.406 |
| IL-1β | |  |  |  |
|  | β (95%CI) | ref | 0.03 (-0.09, 0.15) | -0.03 (-0.15, 0.09) |
|  | *P* |  | 0.637 | 0.639 |
| IL-6 | |  |  |  |
|  | β (95%CI) | ref | -0.03 (-0.20, 0.13) | 0.02 (-0.14, 0.19) |
|  | *P* |  | 0.678 | 0.781 |
| IL-8 | |  |  |  |
|  | β (95%CI) | ref | **0.18 (0.04, 0.32)** | 0.11 (-0.03, 0.25) |
|  | *P* |  | **0.011** | 0.123 |
| CCL2 | |  |  |  |
|  | β (95%CI) | ref | 0.10 (-0.01, 0.20) | 0.02 (-0.09, 0.12) |
|  | *P* |  | 0.071 | 0.716 |
| IL-10 | |  |  |  |
|  | β (95%CI) | ref | 0.01 (-0.12, 0.13) | 0.05 (-0.07, 0.18) |
|  | *P* |  | 0.913 | 0.411 |
| hs-CRP | |  |  |  |
|  | β (95%CI) | ref | -0.002 (-0.09, 0.09) | 0.004 (-0.08, 0.09) |
|  | *P* |  | 0.956 | 0.922 |
| BDNF | |  |  |  |
|  | β (95%CI) | ref | 0.11 (-0.03, 0.25) | **0.15 (0.01, 0.29)** |
|  | *P* |  | 0.122 | **0.038** |

Models were adjusted by age, sex, BMI, hypertension, hyperglycemia, and hyperlipidemia at baseline. CI, confidence interval

**Fig. S3 Correlation plots of association between changes in inflammatory cytokines and standardized cognitive scores in postoperative 3 months**


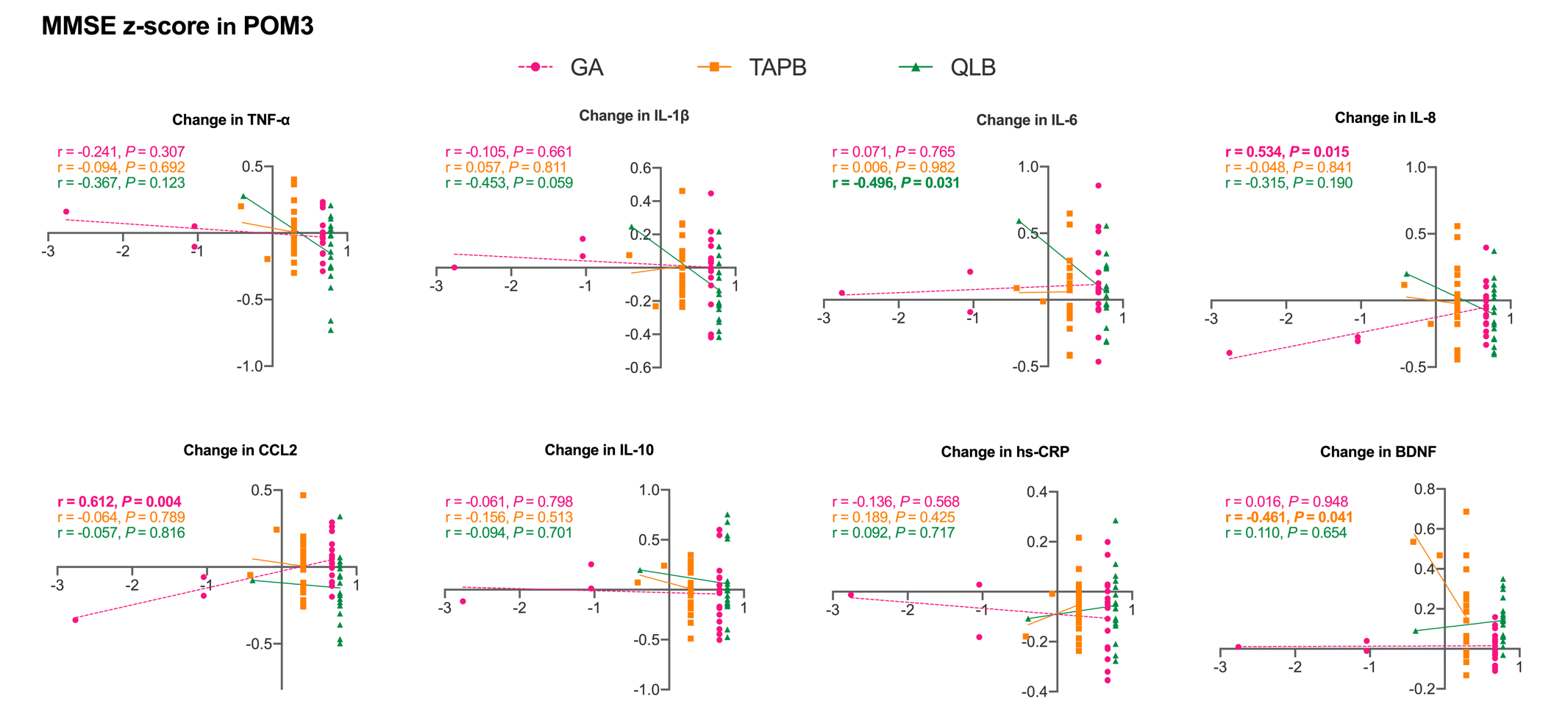


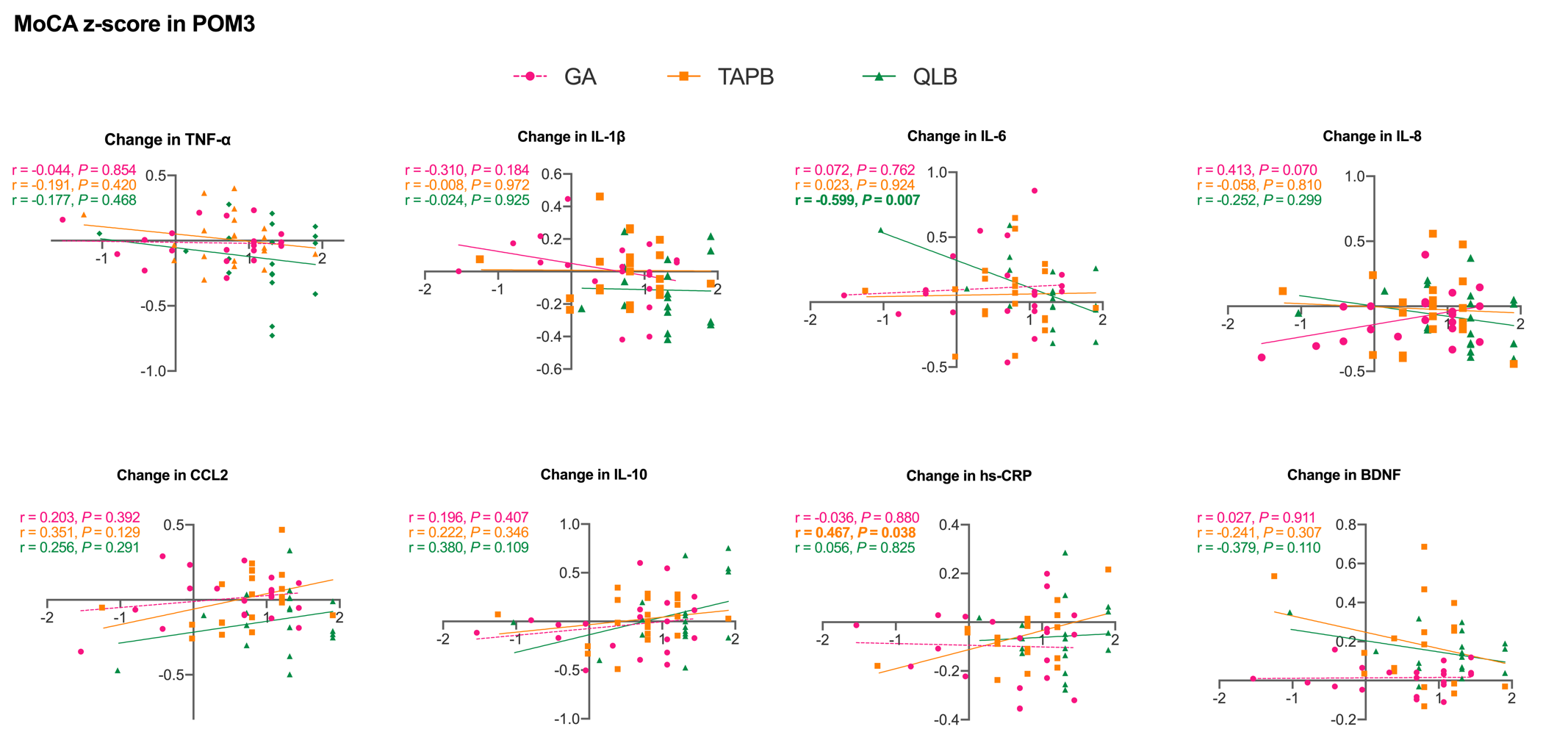


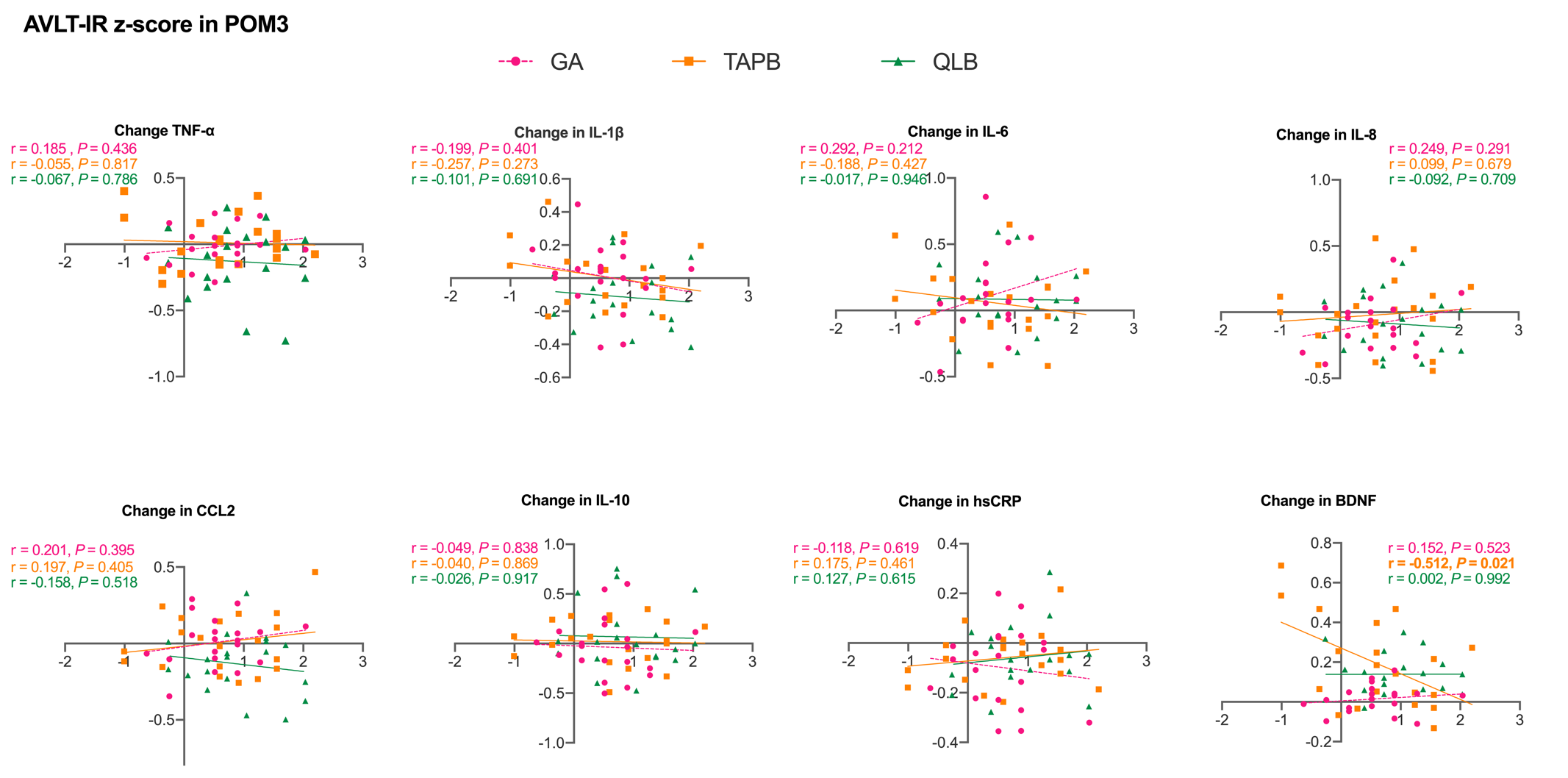


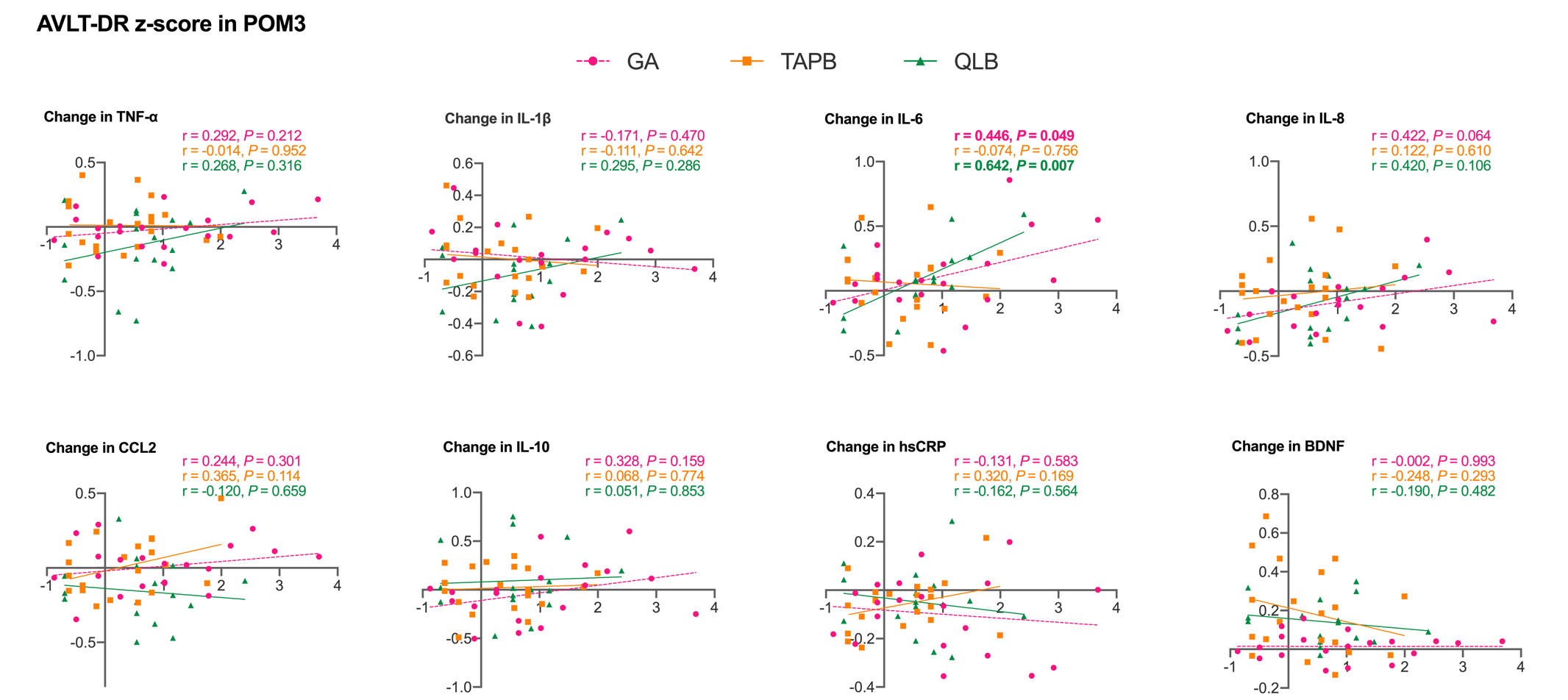


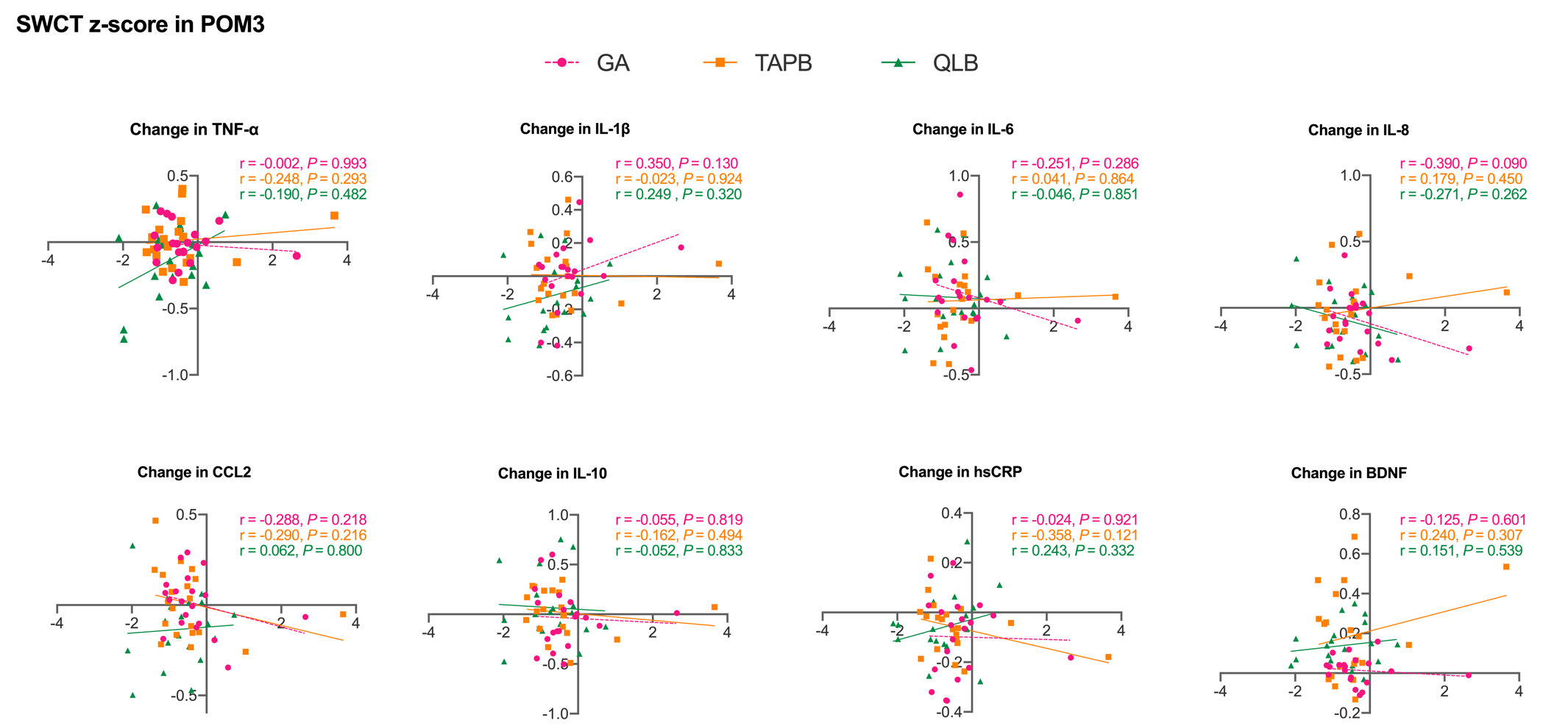


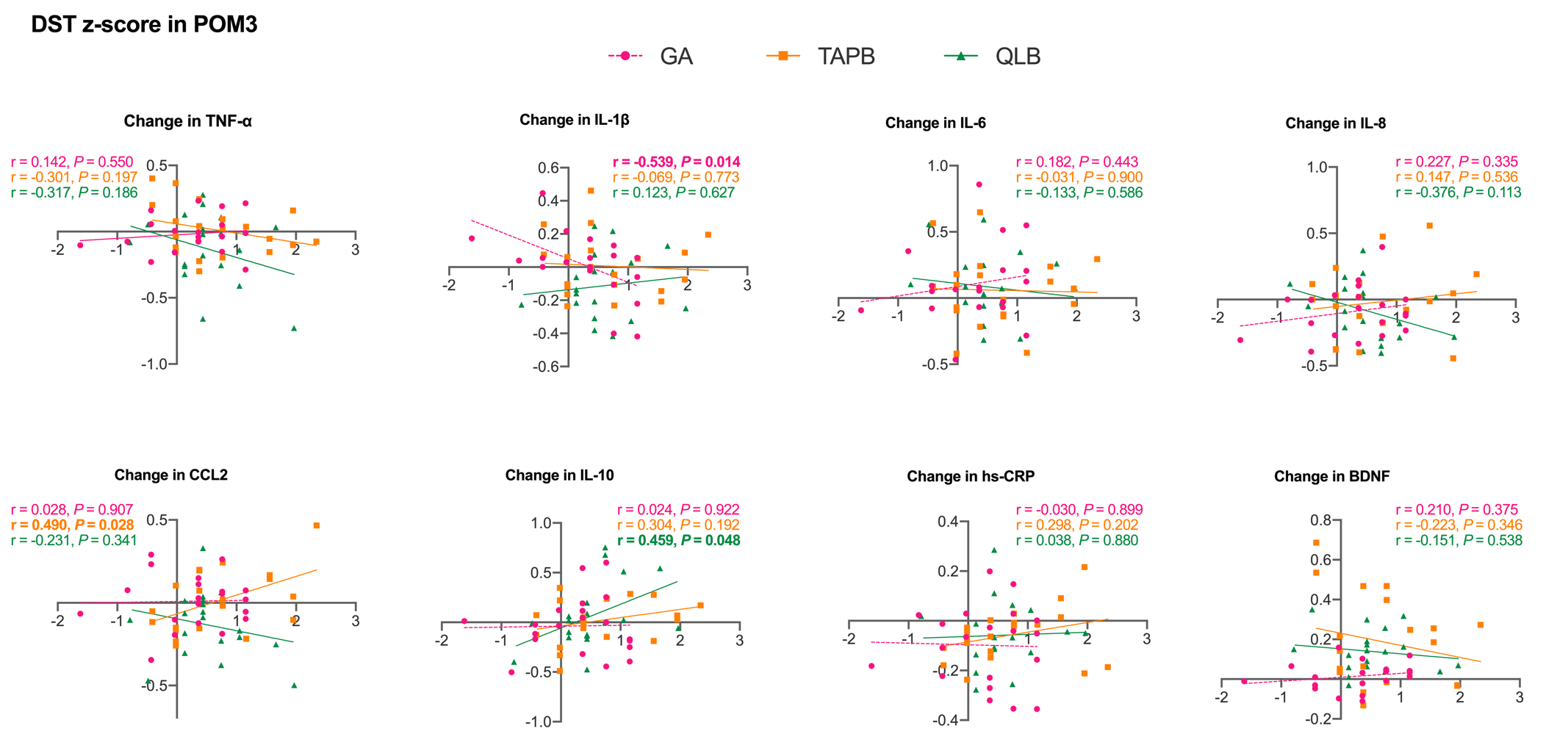

Supplement: Supplementary file 1 — Supplementary file1 (DOCX 37810 KB) [file 11695_2022_6319_MOESM1_ESM.docx]
